# Temporomandibular joint function 10-15 years after mandibular setback surgery and six weeks of intermaxillary fixation

**DOI:** 10.1590/1678-7757-2018-0510

**Published:** 2019-05-30

**Authors:** Mohammedreza Sefidroodi, Ole Kristian Lobekk, Sigbjørn Løes, Elisabeth Schilbred Eriksen

**Affiliations:** 1 University of Bergen, Faculty of Medicine, Department of Clinical Dentistry, Bergen, Norway.; 2 Haukeland University Hospital, Department of Maxillofacial Surgery, Bergen, Norway.

**Keywords:** Intermaxillary fixation, Mandibular setback surgery, Mandibular fracture, Helkimo clinical dysfunction index

## Abstract

Intermaxillary fixation (IMF) is a classic method for immobilization of the mandible after mandibular fractures and corrective surgery. However, it has been suggested that IMF may be a risk for developing temporomandibular joint (TMJ)-related symptoms, especially when applied for longer periods.

**Objective:**

To evaluate the clinical function of TMJs and masticatory muscles 10-15 years after mandibular setback surgery and subsequent six weeks of IMF. The patients' self-reported TMJ and masticatory muscle symptoms were also addressed.

**Methodology:**

Thirty-six patients (24 women and 12 men) treated with intraoral vertical ramus osteotomies and subsequent six weeks of IMF, underwent a clinical examination of TMJs and masticatory muscles 10-15 years after surgery and completed a five-item structured questionnaire reporting subjective TMJ-related symptoms. Mean age by the time of clinical examination was 34.1 years (range 27.2–59.8 years). The clinical outcome was registered according to the Helkimo clinical dysfunction index. Descriptive and bivariate statistics were performed and level of significance was set to 5%.

**Results:**

Mean maximum unassisted mouth opening 10-15 years after surgery was 50.1 mm, (range 38-70 mm, SE 1.2), statistically significantly greater in men compared to women (p=0.004). Mean Helkimo dysfunction group was 1.5 (range 1-3, SE 0.10). Eighty-one percent experienced pain on palpation in either the masseter muscle, temporal muscle or both, and 31% experienced pain when moving the mandible in one or more directions. Thirty-one percent reported pain from palpating the TMJs. In the questionnaire, none reported to have pain during chewing or mouth opening on a weekly or daily basis, but 22% reported difficulties with maximum opening of the mouth.

**Conclusion:**

Ten to fifteen years after mandibular setback surgery the patient's mandibular range of movement is good. Despite clinically recognizable symptoms, few patients reported having TMJ- or masticatory muscle-related symptoms in their daily life.

## Introduction

Intermaxillary fixation (IMF) is a classic method for immobilization of the jaws and is widely used for fracture fixation, and also to some extent after orthognathic surgery. Advancement in osteosynthesis techniques has reduced the need for IMF, allowing for immediate function after treatment. On the other hand, osteosynthesis has the risk of complications such as infections and mental nerve injury.^1^ However, as most patients find IMF uncomfortable, open surgery and fixation with plates and screws has gradually been the treatment of choice for most jaw fractures and after orthognathic surgery. It has been suggested that IMF, especially when applied for longer periods, may be a risk for developing temporomandibular joint (TMJ)-related symptoms.^2^ These findings have been explained by the transient muscular atrophy following the enforced jaw hypo-mobilization. Temporary advantages concerning postoperative mobility and TMD symptoms using rigid osteosynthesis compared with IMF have been reported,^3^ while other studies have failed to find any difference between the two fixation methods.^4^ It is suggested that orthognathic surgery itself, i.e. without IMF, has little or no adverse effect on the temporomandibular joint and mandibular mobility,^5^ although certain subgroups may be at risk.^6^

The aim of this study was to evaluate the clinical function of the temporomandibular joint and masticatory muscles 10-15 years after mandibular setback surgery and subsequent six weeks of IMF. The patients' self-reported symptoms from the TMJs and masticatory muscles were also addressed.

## Methodology

### Patients

The participants in this study were previous patients with genuine mandibular prognathism operated with intraoral vertical ramus osteotomy and subsequent IMF for six weeks from January 1998 to December 2002. Patients who had additional maxillary surgery or genioplasty were not included. The treatment was planned and coordinated by a regional orthognathic surgery team. The surgeries were performed at a university hospital. Pre- and post-surgical orthodontic treatment had been performed in all patients.

The patients were contacted by mail and invited to attend a 10-15 years follow-up examination during the year 2012. Out of the 84 patients operated with the IVRO procedure from January 1998 to December 2002, thirty-seven patients (44%) agreed to participate in the study. Thirty-nine patients (46.6%) did not reply to the invitation, six patients (7.1%) were busy during the time the data collection took place, and two patients (2.4%) did not want to participate. One of the 37 participants was excluded due to a history of mandibular fracture during the follow-up period. The final study group consisted of thirty-six patients (24 females and 12 males). Their mean age at the follow-up examination was 34.1 years (range 27.2–59.8 years) (Table 1). The mean time between surgery and long-term follow-up examination was 12.5 years (range 9.7-14.5 years). Written informed consent was collected from all the participants prior to enrollment. The study was given ethical approval by the regional ethics committee (2011/1604/ REK Vest) and was conducted in accordance with the Declaration of Helsinki.

**Table 1:** Age distribution during clinical examination 10-15 years after surgery

	Mean	SE	95% CI	Min	Max
Men (n=12)	34.8	0.8	33.2-36.5	30.3	38.8
Women (n=24)	33.7	1.5	30.6-36.8	27.2	59.8
All (n=36)	34.1	1.0	32.0-36.2	27.2	59.8

SE= standard error; CI= confidence interval

### Methods

The long-term follow-up consultation included examination of the masticatory muscles and TMJs according to the Helkimo clinical dysfunction index.^7^ This index includes an evaluation of TMJ function, range of movement, occasional pain during function, and pain upon palpation of the joint or masticatory muscles.^7^ The deep and superficial parts of the masseter muscle, anterior and posterior part of the temporal muscle and its attachment to the coronoid process, and the lateral and medial pterygoid muscles were subjects to examination. According to the criteria for the Helkimo index, only muscles that are clearly tender on palpation are to be noted as painful.^7^ The patient has to produce a response either verbally, by stating pain, or by a palpebral reflex. The clinical examination was performed by one examiner. Based on the severity of the symptoms, a dysfunction score was calculated for each participant. The range of the dysfunction score is 0 to 25 points. The dysfunction score was further used to classify the patients into groups representing no, mild, moderate or severe dysfunction. The clinical dysfunction group 1 includes patients with mild dysfunction (dysfunction score 1-4 points) and further represents the clinical dysfunction index 1 (D_i_I). Clinical dysfunction group 2 (dysfunction score 5-9 points) includes patients with moderate dysfunction which further represent the clinical dysfunction index 2 (D_i_II). Patients with severe dysfunction are those with a dysfunction score of 10-25 points, corresponding to the clinical dysfunction groups 3-5 and the clinical dysfunction index 3 (D_i_III).

Prior to the long-term follow-up examination, the patients completed a structured questionnaire. The questionnaire included five questions concerning pain and symptoms from the TMJs and masticatory muscles: pain during chewing/mouth opening, joint sounds such as crepitation and/or clicking, restricted mouth opening, and jaw fatigue. One of the participants did not return the questionnaire.

### Statistical methods

Descriptive statistics were used to report age and gender distribution among the participants, as well as to report the clinical results and the responses to the questionnaires. Distribution of the continuous variables were tested with the Shapiro-Wilk test. Differences between genders for the measurements on jaw mobility were analyzed with the two-sample *t*-test, and the Kruskal-Wallis test was used for the Helkimo dysfunction score. Fisher's exact tests were used to test for differences between genders for dichotomized variables. Level of significance was set to 5%. The statistics application software STATA/IC 14.1 (StataCorp LP, College Station, TX, USA) was used for the analyses.

## Results

The results from the clinical examination 10-15 years after surgery are listed according to the Helkimo index (A-F):

### A. Range of movement

Mean maximum unassisted mouth opening was 50.1 mm, (range 38-70 mm, SE 1.2), and statistically significantly wider in men compared to women (*p*=0.004). Mean maximum lateral movement to the right was 10.2 mm (range 7-15 mm, SE 0.3). Mean maximum lateral movement to the left was 10.1 mm (range 4-14 mm, SE 0.3). Female patients had significantly greater mean maximum lateral movement to the left compared to male patients (*p*=0.02). Mean maximum protrusion was 8.1 mm (range 4-12.5 mm, SE 0.3) (Table 2A).

**Table 2:** Results from clinical examination 10-15 years after surgery listed according to Helkimo clinical dysfunction index

**A. Range of movement**						
	**Mean**	**SE**	**95% CI**	**Min**	**Max**	**n**
Max mouth opening (mm)	50.1	1.2	47.7 - 52.4	38.0	70.0	36
Max right laterotrusion (mm)	10.2	0.3	9.5 - 10.9	7.0	15.0	36
Max left laterotrusion (mm)	10.1	0.3	9.3 - 10.8	4.0	14.0	36
Max protrusion (mm)	8.1	0.3	7.5 - 8.8	4.0	12.5	36
B. Function of the TMJ						
	**Yes**		**No**		**Total**	
	**n**	**%**	**n**	**%**	**n**	
Straight opening and closing path	29	80.6	7	19.4	36	
Crepitation	2	5.6	34	94.4	36	
Clicking	12	33.3	24	66.7	36	
Lateral deviation ≥ 2 mm during opening/closing	7	19.4	29	80.6	36	
Locking during movement	1	2.8	35	97.2	36	
Luxation during movement	0	0	36	100.0	36	
**C. Muscle pain**						
	**Yes**		**No**		**Total**	
	**n**	**%**	**n**	**%**	**n**	
Deep masseter	22	61.1	14	38.9	36	
Superficial masseter	23	63.9	13	36.1	36	
Masseter total	26	72.2	10	27.8	36	
Posterior temporal muscle	11	30.6	25	69.4	36	
Anterior temporal muscle	9	25.0	27	75.0	36	
Temporal muscle on the coronoid process	6	16.7	30	83.3	36	
Temporal muscle total	15	41.7	21	58.3	36	
Lateral pterygoid muscle	36	100.0	0	0.0	36	
Medial pterygoid muscle	25	69.4	11	30.6	36	
**D. Pain on palpation of the TMJs**						
	**Yes**		**No**		**Total**	
	**n**	**%**	**n**	**%**	**n**	
Total	11	30.6	25	69.4	36	
Lateral	10	27.8	26	72.2	36	
Posterior	1	2.8	35	97.2	36	
**E. Pain during jaw movements**						
	**Yes**		**No**		**Total**	
	**n**	**%**	**n**	**%**	**n**	
Pain on any movement of the mandible	11	30.6	25	69.4	36	
Pain on max opening	10	27.8	26	72.2	36	
Pain on right laterotrusion	3	8.3	33	91.7	36	
Pain on left laterotrusion	2	5.6	34	94.4	36	
Pain on protrusion	1	2.8	35	97.2	36	

Max: maximum, min: minimum, mm: millimetre, CI: confidence interval, TMJ: temporomandibular joint

### B. Function of the TMJ

Eighty-one percent of the patients had a straight opening and closing path, while the remaining 19% had lateral deviation during opening or closing of the mouth. Clicking in the joint, either uni- or bilaterally, was registered in 33% of the patients (Table 2B).

### C. Muscle pain

All patients experienced pain on palpation of one or more masticatory muscles, either uni- or bilaterally. Seventy-two percent of the patients had 1-3 muscles that were painful upon palpation, while 28% of the patients felt pain on palpation in four or more palpated muscles (Table 2C). Only muscles with clear and significant tenderness were recorded, as specified by Helkimo. Patients with masseter or temporal muscle tenderness did not show any reduction in mouth opening (data not shown).

### D. Pain on palpation of the TMJs

Thirty-one percent of the patients reported pain on palpation of the TMJ either uni- or bilaterally. Twenty-eight percent of patients experienced pain on palpation on the lateral aspect of the condyle, while one patient reported pain when the condylar head was palpated in the posterior area via the auditory canal (Table 2D).

### E. Pain during jaw movements

The majority of patients (69.4%) reported no pain on any movement of the mandible. Ten patients (27.8%) experienced pain on maximum opening of the mouth, and four patients (11.1%) reported pain during lateral movements or protrusion (Table 2E).

### F. Helkimo clinical dysfunction score

The mean Helkimo dysfunction score was 4.0 (range 1-10, SE 0.45) (Table 3).

**Table 3:** Number of patients classified after the Helkimo clinical dysfunction score, group and index

F	n	G	H
0	0	G0 (0)	Di0 (0)
1	7	G1 (21)	DiI (21)
2	7		
3	6		
4	1		
5	4	G2 (13)	DiII (13)
6	2		
7	6		
8	1		
9	0		
10	2	G3 (2)	DiIII (2)
11 - 13	0		
15 - 17	0	G4 (0)	
20 - 25	0	G5 (0)	

F= Helkimo clinical dysfunction score (Sum A+B+C+D+E)0-25; n= number of patients with respective score 0-25; G= Helkimo clinical dysfunction groups G1-5 and number of patients in each group; H=Helkimo clinical dysfunction index DiI-III and number of patients

### G. Helkimo clinical dysfunction group

Mean clinical dysfunction group was 1.47 (range 1-3, SE 0.10). Ninety-four percent of the patients were diagnosed as being in dysfunction group one or two. None of the patients had a clinical dysfunction score representing the two most severe dysfunction groups (group 4 or 5) (Table 3).

### H. Clinical dysfunction index (D_i_)

None of the patients were placed in D_i_0. Most of the patients were placed in the dysfunction index D_i_I (21 patients) or D_i_II (13 patients). Only 2 patients fulfilled the requirements of D_i_III (Table 3).

### Questionnaire

The responses to the questionnaire are presented in Table 4. On a weekly or daily basis, none of the patients reported any problem with pain while chewing or opening the mouth, but eight patients reported weekly (n=6) or daily (n=2) difficulties with maximum opening of the mouth. The two patients who reported difficulties with maximum mouth opening on a daily basis had maximum opening capacity measured to 38.0 mm and 47.5 mm at the clinical examination. Four patients reported to have clicking in the TMJ at least once a week, and three patients experienced clicking in the TMJ every day.

**Table 4:** Responses to the questionnaire (n=35)

	Never		Rarely		Weekly		Daily		Missing	
	n	%	n	%	n	%	n	%	n	%
Pain during chewing/mouth opening	23	65.7	12	34.3	0	0	0	0.0	0	0.0
Crepitation sounds from TMJ	23	65.7	8	22.9	1	2.9	0	0.0	3	8.6
Clicking sounds from TMJ	16	45.7	9	25.7	4	11.4	3	8.6	3	8.6
Difficult to fully open the mouth	20	57.1	6	17.1	6	17.1	2	5.7	1	2.9
Fatigue in the jaws	12	34.3	17	48.6	6	17.1	0	0.0	0	0.0

TMJ: temporomandibular joint

## Discussion

Temporomandibular disorders (TMD) have a multifactorial etiology, and limited knowledge exists on IMF-induced long term TMJ symptoms. Dervis, et al.^2^ (2002) reported increased TMD symptoms and reduced jaw mobility after the use of IMF. These findings were however reported to be temporary and reversed after 1-2 years. Other studies have reported reduction of TMJ sounds and pain after surgery using IVRO followed by a period of maxillomandibular fixation^8^ ^–^ ^11^. Patients with need of orthognathic surgery may have increased risk for TMJ-symptoms due to occlusal instability,^12^ and advancement as well as setback surgery has been reported to improve TMD symptoms.^13^ ^,^ ^14^ A comparison between vertical ramus osteotomy (VRO) and sagittal split osteotomy (SSO) in a study including more than 1500 patients showed that preoperatively, 44% of VRO- and 44% of SSO-patients reported subjective TMD symptoms. Postoperatively, only 22% of VRO-treated patients reported subjective symptoms of TMD while 35% of SSO-treated patients reported symptoms.^15^ Westermark, et al.^15^ (2001) reported that IMF after IVRO reduces the maxillomandibular opening capacity compared to patients treated with SSO. However, the reduction was temporary and resolved within 6 months after surgery.^16^

The mean Helkimo dysfunction group in our study was 1.5, which is between mild and moderate dysfunction. A significant contributing factor to this result was muscle pain during direct palpation of the masticatory muscles. All of our patients reported pain upon palpation of the lateral pterygoid muscles. According to Türp, et al.^17^ (2001), palpation of the lateral pterygoid muscles may produce false positive findings among healthy individuals due to its low validity and reliability. Only one palpable masticatory muscle site is required to be awarded one point in the Helkimo clinical dysfunction index, and false positive findings from palpating the lateral pterygoid muscle may cause an over-representation of patients in the muscle pain category. According to Helkimo, only muscles that are clearly tender to palpation are to be noted as painful either by a verbal response from the patient, confirming pain, or by a palpebral reflex.^7^ There may be subjective variations in interpretation of pain intensity, and we are aware that the Helkimo clinical dysfunction index has its limitations compared to more comprehensive indices like the RDC/TMD. However, the Helkimo index is simple to conduct and was therefore chosen for this study. The clinical examinations of masticatory muscles and TMJs were performed by a single examiner, without calibration with other clinicians. The examiner was a general dental practitioner. Interpretation of the tenderness is a subjective matter, and patient's response may also vary according to time and expectations. However, self-reported symptoms were significantly lower compared to what was registered during clinical examination. These findings indicate that despite a clinically recognizable tenderness to palpation, it is not necessarily considered a problem for the patients in their daily life. During palpation of the condylar head, one patient reported pain upon posterior palpation, while 10 patients reported pain during lateral palpation. Conclusively did 69% of the patients not report any pain from palpating the TMJs.

A cross-sectional study on the prevalence of mandibular dysfunction in a randomly selected adult Swedish population in the year 2003 found that 4% had severe dysfunction (D_i_III) according to the Helkimo index.^18^ The two patients (5.6%) in the present study diagnosed as having severe dysfunction according to the Helkimo index had both a dysfunction score of 10. This is the lowest score representing severe dysfunction. It is difficult to say if the slightly higher prevalence of patients with dysfunction index III in the present study is a result of the orthognathic treatment they received 10-15 years earlier, if the patients had a preexisting TMD before treatment, or if it was acquired regardless of treatment. The lack of comparable pre-treatment clinical data concerning masticatory muscle- and TMJ-related symptoms is a limitation of this study. Some pre-treatment and post-operative clinical data were available in the patient archive, but the data were not comparable with the data collected at the long term follow-up examination.

Even though several studies have shown that maximum mouth opening is reduced after orthognathic surgery,^8^ ^,^ ^19^ ^,^ ^20^ the results in the present study indicate that mandibular range of movement 10-15 years after surgery is within normal values according to the consensus judgement of the Permanent Impairment Conference.^21^

Almost one third of the patients reported pain during movement of the mandible in one or more direction at the clinical examination. However, the 34.3% of the patients who reported on the questionnaire to have pain during chewing or mouth opening reported that the pain occurred rarely. None of the patients reported pain during chewing or mouth opening on a daily or weekly basis, hence pain during jaw movements does not seem to be a problem for patients 10-15 years after surgery.

In conclusion, the results of this study show that 10-15 years after mandibular setback surgery and subsequent six weeks of IMF the patients' mandibular range of movement is good. Despite clinically recognizable symptoms, few patients reported to have TMJ- or masticatory muscle-related symptoms in their daily life.
